# Emphysematous gastritis

**DOI:** 10.1002/ccr3.5094

**Published:** 2021-11-16

**Authors:** Ryosuke Takase, Naoko Fukuda, Osamu Sui, Hideharu Hagiya

**Affiliations:** ^1^ Department of General Medicine Okayama University Graduate School of Medicine, Dentistry, and Pharmaceutical Sciences Okayama Japan; ^2^ Department of Internal Medicine Marugame Medical Center Marugame Kagawa Japan; ^3^ Department of Radiology Marugame Medical Center Marugame Kagawa Japan

**Keywords:** emphysematous gastritis, phlegmonous gastritis, portal venous gas

## Abstract

An 89‐year‐old woman who had type 2 diabetes mellitus suddenly presented with abdominal pain. Abdominal computed tomography showed thickening of the wall and intramural gas in the gastric hilum, suggesting emphysematous gastritis. The patient underwent treatment with a proton‐pump inhibitor and broad‐spectrum antibacterial agents.

## Graphical abstract

Emphysematous gastritis caused by gas‐producing bacteria have been reported to be cured without surgery by early and appropriate medical treatment, which should be widely recognized by general physicians.

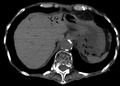

An 89‐year‐old woman who had type 2 diabetes mellitus and chronic kidney disease had been admitted to our hospital for rehabilitation. She suddenly presented with abdominal pain, vomiting, and hypotension. Abdominal computed tomography showed thickening of the wall and intramural gas in the gastric hilum, suggesting emphysematous gastritis, along with portal gas in the left lobe of the liver (Figure 1A). The patient underwent treatment with a proton‐pump inhibitor and broad‐spectrum antibacterial agents. Blood cultures taken prior to the initiation of antimicrobial therapy were negative. Two weeks after the onset, endoscopy showed post‐hemorrhagic changes in the gastric mucosa on the upper gastric bends (Figure 1B). The patient gradually recovered, resumed oral intake, and was discharged.

**FIGURE 1:**
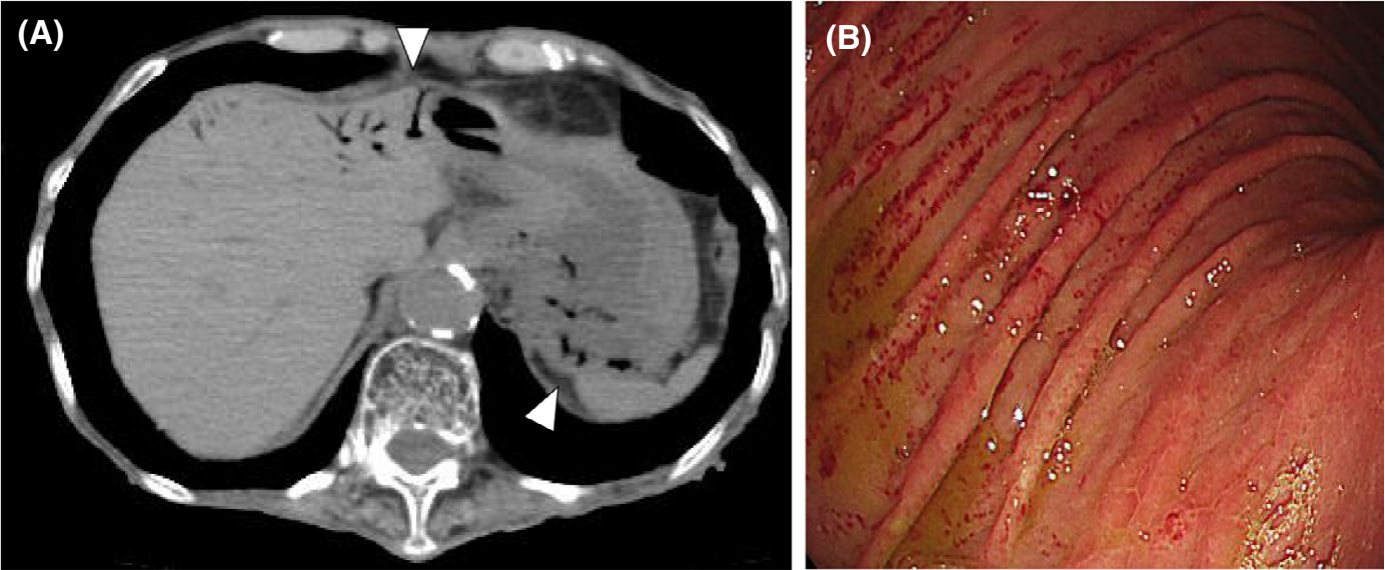
Abdominal computed tomography showing emphysematous gastritis and portal venous gas (A) and endoscopic findings (B)

Emphysematous gastritis is caused by gas‐producing bacteria, the mortality rate has been reported to be 55.3%.^1^ As in our case, diabetes mellitus, malnutrition, and renal failure are risk factors for the disease.^1^ Although it is important to differentiate it from gastric emphysema, cystic emphysema in the gastric wall is a characteristic of emphysematous gastritis on a CT scan.^2^ When a patient with the abovementioned risk factors has sudden abdominal pain, emphysematous gastritis should be included in differential diagnosis.

## CONFLICTS OF INTEREST

The authors state that there are no conflicts of interest to declare.

## AUTHOR CONTRIBUTION

RT contributed to data collection, literature review, consent taking, and manuscript writing. NF and OS contributed to the clinical management of the patient. HH organized writing the manuscript.

## ETHICAL APPROVAL

Written consent was obtained from the patient.

## CONSENT

All the mentioned authors obtained for publication.

## Data Availability

Data sharing is not applicable to this article as no datasets were generated or analyzed during the current study.
